# High and Increasing *Oxa-51* DNA Load Predict Mortality in *Acinetobacter baumannii* Bacteremia: Implication for Pathogenesis and Evaluation of Therapy

**DOI:** 10.1371/journal.pone.0014133

**Published:** 2010-11-30

**Authors:** Yu-Chung Chuang, Shan-Chwen Chang, Wei-Kung Wang

**Affiliations:** 1 Department of Internal Medicine, National Taiwan University Hospital, Tapei, Taiwan; 2 Department of Internal Medicine, National Taiwan University Hospital, Yun-Lin Branch, Douliou City, Taiwan; 3 Institute of Microbiology, College of Medicine, National Taiwan University, Taipei, Taiwan; University of Liverpool, United Kingdom

## Abstract

**Background:**

While quantification of viral loads has been successfully employed in clinical medicine and has provided valuable insights and useful markers for several viral diseases, the potential of measuring bacterial DNA load to predict outcome or monitor therapeutic responses remains largely unexplored. We tested this possibility by investigating bacterial loads in *Acinetobacter baumannii* bacteremia, a rapidly increasing nosocomial infection characterized by high mortality, drug resistance, multiple and complicated risk factors, all of which urged the need of good markers to evaluate therapeutics.

**Methods and Findings:**

We established a quantitative real-time PCR assay based on an *A. baumannii*-specific gene, *Oxa-51*, and conducted a prospective study to examine *A. baumannii* loads in 318 sequential blood samples from 51 adults patients (17 survivors, 34 nonsurvivors) with culture-proven *A. baumannii* bacteremia in the intensive care units. *Oxa-51* DNA loads were significantly higher in the nonsurvivors than survivors on day 1, 2 and 3 (*P* = 0.03, 0.001 and 0.006, respectively). Compared with survivors, nonsurvivors had higher maximum *Oxa-51* DNA load and a trend of increase from day 0 to day 3 (*P*<0.001), which together with Pitt bacteremia score were independent predictors for mortality by multivariate analysis (*P* = 0.014 and 0.016, for maximum *Oxa-51* DNA and change of *Oxa-51* DNA, respectively). Kaplan-Meier analysis revealed significantly different survival curves in patients with different maximum *Oxa-51* DNA and change of *Oxa-51* DNA from day 0 to day 3.

**Conclusions:**

High *Oxa-51* DNA load and its initial increase could predict mortality. Moreover, monitoring *Oxa-51* DNA load in blood may provide direct parameters for evaluating new regimens against *A. baumannii* in future clinical studies.

## Introduction

The successful employment of quantitative PCR to measure viral load in clinical medicine has not only advanced our understanding of pathogenesis of many viral diseases but also provided useful markers for predicting outcome and/or monitoring therapeutic responses [1]. The possibility of measuring bacterial DNA load to predict outcome or evaluate therapeutics in bacterial diseases, especially those with high mortality, antimicrobial resistance, multiple and complicated risk factors, has been raised but never been fully explored [1], [2].

*Acinetobacter baumannii* (*A. baumannii*), a nonfermenting Gram-negative aerobic coccobacillus which can survive for prolonged periods in various environmental conditions, is an increasingly important nosocomial pathogen [3]. Among the *A. calcoaceticus- A. baumannii* complex, which included genospecies 1, 2, 3 and 13TU, *A. baumannii* (genospecies 2) is primarily associated with human diseases [4]. Nosocomial infection caused by *A. baumannii* has increased substantially, especially among patients in the ICU [3]–[6] or immunocompromized hosts [3], [4], [6]. It is a life-threatening opportunistic infection among critical ill patient [7], [8]. Patients with *A. baumannii* infection have been reported to be associated with higher mortality and longer hospital stay compared with other infections [3], [7], [8]. In addition to pneumonia, bacteremia is the most common focus of *A. baumannii* infection [4]. Due to rapid increase in the drug resistance of *A. baumannii*, the choices of antimicrobial agents were limited [3], [4], [9]. Previous studies have identified multiple and complicated risk factors for the high mortality of *A. baumannii* bacteremia [5], [6], [10]–[15], but none has investigated the roles of bacterial load in the disease outcome.

The relationship between the magnitude of bacteremia and clinical outcome was originally demonstrated by semiquantitative blood culture [16], [17]. The drastic decrease in the sensitivity of blood culture after antimicrobial treatment and the time required for blood culture limited the application of this approach [2], [18]. Recently, several studies using quantitative PCR assays have shown high initial bacterial DNA loads in blood correlated with disease severity and mortality in infection caused by *Streptococcus pneumoniae, Neisseria meningitides,* and methicillin-resistant *Staphylococcus aureus* (MRSA) [19]–[22]. However, the change in bacterial DNA load during the course of infection and its relationship to disease outcome or therapy remain largely unclear. We reported previously that high levels of *mecA* DNA in blood after 3 and 7 days of antibiotic therapy were associated with mortality in MRSA bacteremia [21], suggesting sequential bacterial DNA load in blood can be used to evaluate therapeutic responses. In this study, we established a quantitative real-time PCR assay by using an *A. baumannii*-specific gene, *Oxa-51*, to measure both the initial and sequential *A. baumannii* loads in blood from patients with *A. baumannii* bacteremia and to investigate the relationships to disease outcome [23].

## Methods

### Patients and blood samples

This prospective observational study was conducted in accordance with the principles expressed in the Declaration of Helsinki at the National Taiwan University Hospital, a medical center with a 2,200-bed capacity in Taiwan. With the approval of the Institutional Review Board (No. 200809045R), adult patients admitted to the medical or surgical ICU between 1 April 2008 and 28 February 2009 were enrolled once a positive blood culture for *A. baumannii* was reported. Blood cultures were taken when clinical symptoms/signs of infection such as fever, shock, etc. were observed. The Board waived the need for informed consent, since this study required no additional blood drawing from the study patients in ICU and all the data were analyzed anonymously (Text S1 and S2). All blood cultures were performed by the Bactec 9240 system (Becton Dickson, Sparks, MD) in the microbiological laboratory. *A. baumannii* complex was identified by classic methods and verified by the Phoenix system (BD Diagnostics, Sparks, MD) [24], followed by a disk diffusion susceptibility test with gentamicin, amikacin, ciprofloxacin, levofloxacin, cefepime, ceftazidime, aztreonam, ticarcillin/clavulanate, meropenem, and ampicillin/sulbactam [25]. The susceptibility of tigecycline was determined by the agar dilution method, in which MIC ≤ 2 µg/mL was considered the break point [26], and that of colistin by E-test [25], [27]. Genospecies *A. baumannii* was defined by *A. baumannii* complex positive for the *Oxa-51* by real-time PCR assay [23]. The course of *A. baumannii* bacteremia in each patient was carefully monitored by clinical parameters and routine laboratory tests. Whole blood samples in EDTA-containing tubes taken before and on the day of reporting *A. baumannii* bacteremia were retrospectively obtained from the central laboratory. After that, blood samples were collected daily for the first week and two to three times a week thereafter.

### Clinical data and definitions

The demographic, clinical, laboratory and culture data for each patient were collected (Tables 1 and 2). The underlying illness was assessed by the Charlson score [28], and clinical severity by the acute physiology and chronic health evaluation II (APACHE II), Pitt bacteremia scores [29], and sequential organ failure assessment score (SOFA) score [30].

**Table 1:** Clinical and laboratory characteristics of study patients with *A. baumannii* bacteremia.

Characteristics^a^	Total (n = 51)	Nonsurvivor group (n = 34)	Survivor group (n = 17)	*P*
Age	61.5 (48.9–74.2)	59.9 (48.0–74.6)	61.5 (49.0–71.7)	0.84
Sex, M/F	33/18	22/12	11/6	1.00
Days of prior hospitalization^b^	18 (10–41)	19.5 (10–47.3)	18 (10–29.5)	0.75
Days of prior ICU stay^b^	10 (0–18)	10 (0.8–16.3)	14 (0–18)	0.80
APACHE II score, ICU admission	26 (20.5–36)	27 (20.5–36)	25.5 (20.3–35)	0.73
Underlying illness				
Charlson score	3 (2–6)	3 (2–6)	4 (2–6)	0.91
Diabetes mellitus	20 (39.2)	11 (32.4)	9 (52.9)	0.23
Congestive heart failure	7 (13.7)	5 (14.7)	2 (11.8)	1.00
Chronic renal insufficiency	16 (31.4)	11 (32.4)	5 (29.4)	1.00
Immunosuppressants^c^	22 (43.1)	18 (52.9)	4 (23.5)	0.07
Leukemia	7 (13.7)	7 (20.6)	0 (0)	0.08
Lymphoma	3 (5.9)	2 (5.9)	1 (5.9)	1.00
Focus of infection				
Primary bacteremia	11 (21.6)	7 (20.6)	4 (23.5)	1.00
Pneumonia	33 (64.7)	24 (70.6)	9 (52.9)	0.23
Intra-abdominal infection	4 (7.8)	2 (5.9)	2 (11.8)	0.59
Surgical site infection	4 (7.8)	4 (11.8)	0 (0)	0.29
Laboratory data on day 0^b^				
White blood cell count (1000/µL)	10.29 (2.42–20.34)	8.36 (0.55–20.64)	12.07 (8.50–19.97)	0.19
Hemoglobin (g/dL)	9.4 (8.5–10.4)	9.2 (8.2–10.5)	9.6 (8.9–10.7)	0.35
Platelet count (1000/µL)	73 (41–134)	56 (19–96.8)	134 (74–275.5)	<0.001
AST^d^ (U/L)	50 (25–95)	67 (30–117)	35 (17.5–58.3)	0.15
Total bilirubin (mg/dL)	2.56 (0.83–9.06)	3 (0.8–25.5)	1.9 (0.7–3.3)	0.32
Creatinine (mg/dL)	1.8 (1.1–3.2)	1.9 (1.1–3.8)	1.7 (1.1–3)	0.81
C-reactive protein (mg/dL)	9.6 (4.6–16.4)	9.8 (5.3–21.1)	8.7 (2.3–12.7)	0.37

a Data are median (interquartile range) for continuous variables and number of cases (percentage) for categorical variables, with two-tailed Mann-Whitney U test for the former and Fisher's exact test for the later.

b Days of hospitalization and ICU stay prior to day 0, which was the day on which the first blood sample positive for *A. baumannii* by culture was drawn.

c Immunosuppressants included antineoplastic therapy within 6 weeks of the onset of *A. baumannii* bacteremia, corticosteroids at a dose ≧ 20 mg of prednisolone daily for at least 2 weeks or 30 mg of prednisolone daily for at least 1 week before a positive blood culture for *A. baumannii*, and other immunosuppressants such as cyclophosphamide [15].

d AST: aspartate aminotransferase.

**Table 2:** Antimicrobial susceptibility and usage, bacteremia score and *Oxa-51* DNA level of study patients with *A. baumannii* bacteremia.

Characteristics^a^	Total (n = 51)	Nonsurvivor group (n = 34)	Survivor group (n = 17)	*P*	Multivariate^f^ odds ratio [95% CI]	*P*
Pitt bacteremia score, on day 0^b^	5 (3–7)	5.5 (4–7.3)	4 (2–6)	0.02	1.55 [0.94–2.57]	0.087
SOFA score^c^	14.5 (11–19)	16 (13.5–19)	10.5 (6–13)	<0.001		
Co-infection	16 (31.4)	10 (29.4)	6 (35.3)	0.75		
Subsequent blood stream infection	2 (3.9)	1 (2.9)	1 (5.9)	1.00		
Antimicrobial resistance to						
Anti-pseudomonas Penicillin	43 (89.6)	29 (87.9)	14 (93.3)	1.00		
Anti-pseudomonas Cephalosporin	45 (88.2)	31 (91.2)	14 (82.4)	0.39		
Carbapenem	38 (74.5)	25 (73.5)	13 (76.5)	1.00		
Aminoglycosides	44 (86.3)	31 (91.2)	13 (76.5)	0.20		
Fluoroquinolones	48 (94.1)	32 (94.1)	16 (94.1)	1.00		
Ampicillin/Sulbactam	31 (60.8)	25 (73.5)	6 (35.3)	0.01		
Tigecycline	10 (20.8)	8 (24.2)	2 (13.3)	0.47		
Colistin	0 (0)	0 (0)	0 (0)			
ERAB^d^	27 (52.9)	22 (64.7)	5 (29.4)	0.04	1.99 [0.21–18.71]^h^	0.55
Antibiotics used in empirical therapy						
Anti-pseudomonas Penicillin	6 (11.8)	2 (5.9)	4 (23.5)	0.09		
Anti-pseudomonas Cephalosporin	16 (31.4)	10 (29.4)	6 (35.3)	0.75		
Carbapenem	26 (51.0)	21 (61.8)	5 (29.4)	0.04		
Fluoroquinolones	12 (23.5)	8 (23.5)	4 (23.5)	1.00		
Ampicillin/Sulbactam	8 (16.7)	5 (14.7)	3 (17.7)	1.00		
Tigecycline	5 (9.8)	4 (11.8)	1 (5.9)	0.65		
Colistin	5 (9.8)	4 (11.8)	1 (5.9)	0.65		
Appropriate empirical therapy^e^	17 (33.3)	12 (35.3)	5 (29.4)	0.76		
Day 3 *Oxa-51* DNA (log/copies/mL)^b^	1.00 (0–3.01)	2.81 (0.59–3.48)	0 (0–1.69)	0.006	2.19 [1.18–4.07]^g^	0.013^g^
Maximum *Oxa-51* DNA (log copies/mL), day 0 – day 3^b^	3.04 (2.30–3.62)	3.38 (2.70–4.07)	2.30 (1.71–2.89)	<0.001	10.25 [1.61–65.46]	0.014
Change of *Oxa-51* DNA, day 0^b^ – day 3 (Δ log copies/mL/day)	0.12 (−0.73–0.76)	0.40 (−0.09–0.97)	−0.77 (−1.25– −0.14)	<0.001	3.17 [1.24–8.12]	0.016

a Data are median (interquartile range) for continuous variables and number of cases (percentage) for categorical variables, with two-tailed Mann-Whitney U test for the former and Fisher's exact test for the later.

b Day 0 was the day on which the first blood sample positive for *A. baumannii* by culture was drawn.

c SOFA: Sequential organ failure assessment. Not included in the final multivariate regression model due to small case numbers (Text S4).

d ERAB: Extensively resistant *A. baumannii.*

e Appropriate empirical therapy is defined when *A.baumannii* is susceptible to at least one antibiotic used within 48 hours of day 0 except aminoglycoside monotherapy (see Methods).

f Multivariate logistic regression model: *n* = 40, adjusted generalized *R* ^2^ = 0.634, estimated area under the ROC curve  = 0.92. Deviance goodness-of-fit (GOF) test *P* = 0.87>0.05 (df  = 36); Pearson GOF test *P* = 0.88>0.05 (df  = 36); Hosmer and Lemeshow GOF test *P* = 0.98>0.05 (df  = 8).

g Discard “Maximum *Oxa-51* DNA” and “Change of *Oxa-51* DNA”, both of which require daily monitoring of *Oxa-51* DNA in blood.

h ERAB was considered in the predicting model of mortality based on the three independent predictors, Pitt bacteremia score, maximum *Oxa-51* DNA and change of *Oxa-51* DNA from day 0 to day 3.

Day 0 was the day on which the first blood sample positive for *A. baumannii* by culture was drawn, and the days after that were designated consecutively. Since the average time of reporting *A. baumannii* bacteremia was 72 h in our study, the period of empirical therapy was defined as from day 0 to day 3. Extensively resistant *A. baumannii* (ERAB) was defined as *A. baumannii* resistant to the commonly used anti-pseudomonas antibiotics (cephalosporins, extended-spectrum penicillins, carbapenems, aminoglycosides, fluoroquinolones) and ampicillin/sulbactam determined by disc diffusion method [15], [25]. Appropriate empirical therapy was defined if *A. baumannii* was susceptible in vitro to at least one antibiotic used within 48 hours from day 0. Aminoglycoside monotherapy without combination of other susceptible antibiotics was considered as inappropriate [9]. For ERAB, the possibly appropriate antibiotics included tigecycline and colistin. Although the MIC breakpoint for tigecycline susceptibility was 2 µg/mL according to the FDA definition for Enterobacteriaceae [26], it was considered as inappropriate therapy if used alone for *A. baumannii* blood stream infection with MIC ≧1 µg/mL [9]. The overall and initial changes of *Oxa-51* DNA load were assessed by the summary statistics for each patient, in which the slopes of linear regression of log *Oxa-51* DNA copies/mL over the whole course and from day 0 to day 3, respectively, were calculated. Co-infection was defined by the presence of pathogens other than *A. baumannii* in blood culture at the same time when the first blood sample positive for *A. baumannii* by culture was drawn. Subsequent blood stream infection was defined by the presence of pathogens other than *A. baumannii* in blood culture within 14 days after the onset of *A. baumannii* bacteremia.

Patients, who fulfilled the definition of *A. baumannii* bacteremia related death including (1) blood culture positive for *A. baumannii* at the time of death, (2) death before resolution of symptoms and signs, and (3) death within 14 days after the onset of *A. baumannii* bacteremia without another explanation, were classified as the nonsurvivor group. Patients who survived the episode were classified as the survivor group.

### DNA extraction

One milliliter of whole blood from patients or *A. baumannii*-spiked whole blood was treated with 3 mL of red blood cell lysis solution (Gentra Systems, Minneapolis, MN) at room temperature for 5 min, followed by centrifugation at 5,000× g for 10 min to obtain the pellets, which were resuspended in enzyme solution (20 mM Tris [pH 8.0], 2 mM EDTA, 1.2% Triton, lysozyme 20 mg/mL) and subjected to DNA extraction using QIAamp DNA minikit (Qiagen, Hilden, Germany) [21]. The final eluate (100 µl) was stored at –20°C until use. Whole blood samples from healthy donors served as negative controls. DNA extraction and PCR were carried out in separate rooms, and precautions were taken to prevent contamination [31].

### Quantitative real-time PCR

The *Oxa-51*-specific primers (F Oxa-51 and R Oxa-51) and probe (P Oxa-51) were designed based on the *Oxa-51* sequence in GenBank and Primer Express software v2.0. Plasmid Oxa-51/pCRII-TOPO, which contained a 431-bp fragment of the *Oxa-51* gene in pCRII-TOPO, was used as the standard and its copy number was calculated based on the concentration determined by spectrophotometry and molecular weight [21], [32] (Text S3). The sequences of F Oxa-51, R Oxa-51 and P Oxa-51 are 5′-TTTAGCTCGTCGTATTGGACTTGA-3′, 5′-CGGAGAACGACTCCTCAT TAAAAA-3′ and 5′-TGGCAATGCAGATA TCGGTACCCAAGTC-3′, respectively. An aliquot of extracted DNA and known copy numbers of *Oxa-51* DNA standards were subjected to real-time PCR. Briefly, a 25 µl reaction mixture containing 12.5 µl of 2x TaqMan universal PCR master mix, 2.5 µl of 10 pmol/µl of each of F Oxa-51, R Oxa-51 and P Oxa-51, *Oxa-51* DNA standards or extracted DNA, and nuclease free-water was subjected to real-time PCR with an ABI Prism 7000/7500 sequence detector (Applied Biosystems, Foster City, CA). The amplification conditions were 50°C for 2 min and 95°C for 10 min, followed by 50 cycles of 95°C for 15 s and 60°C for 1 min, as described previously [21]. A positive result was defined by the cycle number required to reach the threshold, which was 10 times the standard deviation of the mean baseline emission calculated for cycles 3 to 15 [21]. Since 5 µl of the 100 µl DNA eluate, which was derived from 1 mL of whole blood samples, was used in each reaction mixture, the *Oxa-51* DNA copy number per reaction was multiplied by 20 to determine the copy numbers of *Oxa-51* DNA per mL whole blood. The sensitivity of the assay was 20 copies per mL.

### Statistical analysis

Mann-Whitney U test and Fisher's exact test were used to compare continuous variables and categorical variables, respectively, between two groups. Log-rank test and Kaplan-Meier survival curve were used for survival analysis, and the results were verified by multivariate Cox's proportional hazard model. Multivariate logistic regression analysis including all variables such as age, sex, underlying diseases, laboratory data, infection foci, invasive procedure (central catheter, ventilator), appropriate empirical antibiotics and bacterial load was conducted to identify predictors for mortality (Text S4). Data was analyzed using Stata software, version 10 (StataCorp, College Station, Texas).

## Results

### Quantitative real-time PCR assay for *Oxa-51* gene

We first established a standard curve by using increasing copy numbers (5 to 5×10^8^ copies) of the plasmid Oxa-51/pCRII-TOPO in the assay (r = 0.9992, Text S3). We next tested DNA extracted from increasing CFUs (9×10^1^ to 9×10^6^ CFUs) of a previously reported *A. baumannii* isolate 2003I053 [32], which had been spiked with whole blood from a healthy volunteer, and found a linear curve between the CFUs and *Oxa-51* copy numbers (*r* = 0.9942), demonstrating the accuracy of this assay for quantifying *A. baumannii* in whole blood (Text S3). Moreover, the *Oxa-51* copy numbers of DNA extracted from *A. baumannii*-spiked whole blood that had been stored at 4°C for up to 4 days remained stable, suggesting the feasibility of this assay to quantify *A. baumannii* in stored whole blood samples (Text S3).

The specificity of the *Oxa-51* real-time PCR assay was verified by examining DNA extracted from 85 isolates including common nosocomial pathogens such as *E. coli, K. pneumoniae, E. cloacae, P. aeruginosa, S. maltophilia*, *E. faecium* and MRSA, as well as Acinetobacter genospecies 3, 13TU and 2 (*A. baumannii*) in the assay [32]–[35], which detected positive signal only in the DNA extracted from *A. baumannii* but not in those from other isolates.

### Study Patients

Table 1 summarizes the clinical and laboratory characteristics of the 51 patients with *A. baumannii* bacteremia, including 34 nonsurvivors and 17 survivors. They were predominantly males with a median age of 61.5 years, and were all critically ill with high APACHE II score (median: 26). There was no difference between the two groups in the APACHE II or Charlson score, the number of days of hospitalization and of ICU stay prior to *A. baumannii* bacteremia, individual underlying disease such as leukemia or lymphoma, immunosuppressant, foci of infection, or initial laboratory data except lower platelet counts in the nonsurvivors. Compared with the survivors, the nonsurvivors had higher Pitt bacteremia score and SOFA score on day 0, and higher frequency of ERAB and resistance to ampicillin/sulbactam (*P* = 0.02 and *P*<0.001, Mann-Whitney U test, two tailed; *P* = 0.04 and 0.01, Fisher's exact test, respectively) (Table 2). There was no difference in the co-infection, subsequent blood stream infection, antimicrobial resistance to other groups of antibiotics, antibiotics used during empirical period (except carbapenem) or appropriate empirical therapy between the two groups (Table 2 and Table S1).

### Higher levels of *Oxa-51* DNA in nonsurvivors than survivors

**Figure 1:**
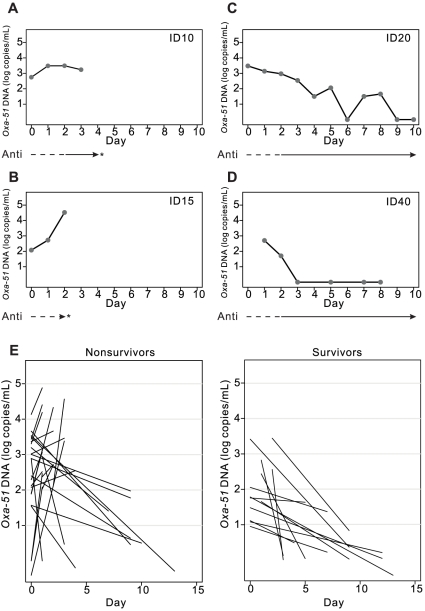
Sequential *Oxa-51* DNA loads in blood during the course of *A. baumannii* bacteremia. Sequential whole blood samples from 2 nonsurvivors (A, B) and 2 survivors (C, D) with *A. baumannii* bacteremia were subjected to DNA extraction and the *Oxa-51* real-time PCR assay. The lines beneath indicate inappropriate (—) and appropriate (—) antibiotic therapy, and the asters indicate death. (E) Overall change of *Oxa*-51 DNA load in blood for patients with *A. baumannii* bacteremia (27 nonsurvivors, 13 survivors) assessed by the slope of the linear regression of log *Oxa-51* DNA copies per mL plotted against time.

We then employed the real-time PCR assay to quantify *Oxa-51* DNA in 318 sequential blood samples from 51 patients. We first examined sequential *Oxa-51* DNA loads over time in 4 patients, including 2 nonsurvivors and 2 survivors. As shown in Figure 1, a decreasing trend of the *Oxa-51* DNA level was found in the survivors, whereas continuously high levels of *Oxa-51* DNA were found in the nonsurvivors. To investigate the overall change of *Oxa-51 DNA* load during the course of infection, we analyzed the slope of *Oxa-51* DNA load. While all survivors had a decline in *Oxa*-51 DNA over time, more than half of the nonsurvivors showed an upward slope at death (*P* = 0.01, Mann-Whitney U test, two tailed) (Figure 1E).

**Figure 2:**
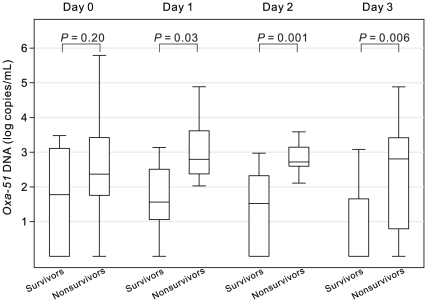
Initial *Oxa-51* DNA load from day 0 to day 3 in patients with *A. baumannii* bacteremia (34 nonsurvivors, 17 survivors). Data are presented as median (line), interquartile range (box) and range.

**Figure 3:**
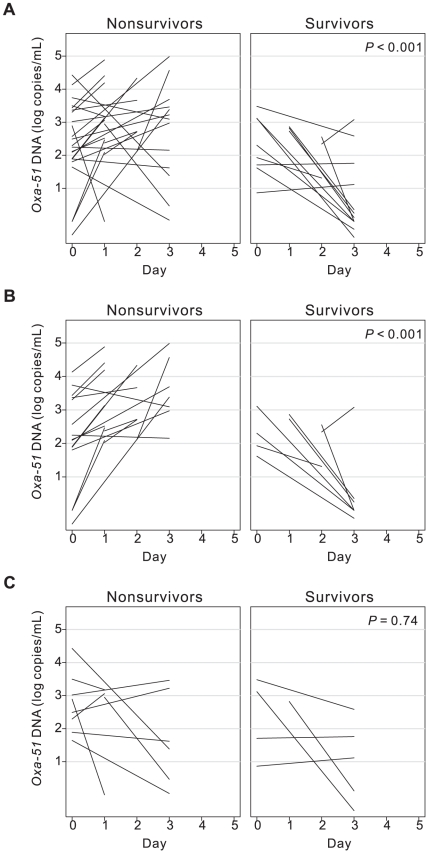
Initial change of *Oxa-51* DNA load from day 0 to day 3 in patients, with *A. baumannii* bacteremia (27 nonsurvivors, 13 survivors) (A), and among subgroups including those receiving inappropriate (B) and those receiving appropriate (C) empirical antibiotics.

We next focused on initial *Oxa-51* DNA loads from day 0 to day 3, which corresponded to the period of empirical therapy. The levels of Oxa-51 DNA were significantly higher in the nonsurvivors than survivors on day 1, day 2 and day 3 (*P* = 0.03, 0.001 and 0.006, respectively, Mann-Whitney U test, two tailed) (Figure 2), so was the maximum *Oxa-51* DNA between day 0 and day 3 (Table 2) [20]. We then examined the change of *Oxa-51* DNA load from day 0 to day 3. As shown in Figure 3A, a trend of increase was found in the majority of nonsurvivors, whereas a trend of decrease in most survivors (median 0.40 vs. −0.77 Δ log copies/mL/day, *P*<0.001, Mann-Whitney U test, two tailed). To further investigate whether appropriate empirical antibiotics affect the *Oxa-51* DNA change during this period, we analyzed subgroups of patients. Among those receiving inappropriate empirical antibiotics, nearly all nonsurvivors showed increasing *Oxa-51* DNA compared with survivors (*P*<0.001, Mann-Whitney U test, two tailed) (Figure 3B). Among those receiving appropriate empirical antibiotics, there was no difference in the initial *Oxa-51* DNA change between the two groups (Figure 3C). Interestingly, the nonsurvivors receiving appropriate empirical antibiotics showed a trend of no or less increase in *Oxa-51* DNA compared with those receiving inappropriate empirical antibiotics (*P*<0.001, Mann-Whitney U test, two tailed) (left panels, Figures 3B and 3C).

### Predictors for mortality

Multivariate logistic regression analysis including all the variables in Tables 1 and 2 revealed that Pitt bacteremia score, maximum *Oxa-51* DNA and change of *Oxa-51* DNA from day 0 to day 3 were three independent predictors for mortality (Table 2). Excluding “maximum *Oxa-51* DNA” and “change of *Oxa-51* DNA”, which required daily monitoring, the single day measurement, *Oxa-51* on day 3, was the most potent independent predictor for those who survived more than 3 days (odds ratio [OR], 2.19; 95% confidence interval [CI], 1.18–4.07; *P* = 0.013). The goodness-of-fit (GOF) test suggested a good fit of the multivariate logistic regression model to binary data and thus a strong prediction (Text S4). Moreover, the predicting model based on the three independent predictors had a positive predictive value of 89.3% and a negative predictive value of 83.3% with the estimated area under receiver operating characteristic (ROC) curve of 0.9202, which was higher than that of 0.6581 based on Pitt bactermia score alone.

### Kaplan-Meier survival analysis

**Figure 4:**
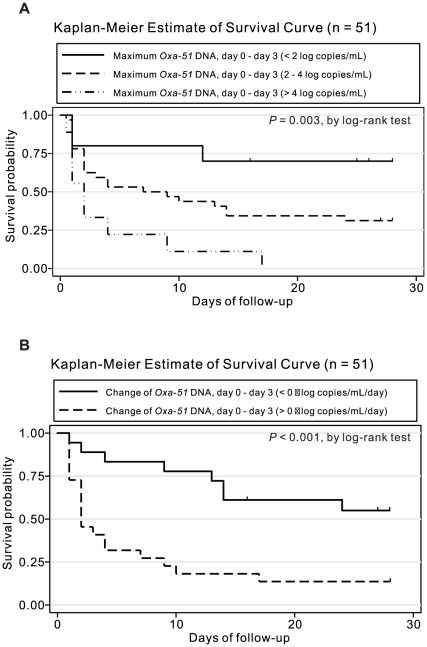
Kaplan-Meier survival curves in patients with *A. baumannii* bacteremia. Comparison among subgroups with different maximum *Oxa-51* DNA load (A) and increased or decreased *Oxa-51* DNA (B) from day 0 to day 3.

Based on the maximum *Oxa-51* DNA from day 0 to day 3, three groups of patients had different survival curves. All patients with high maximum *Oxa-51* DNA (>4 log copies/mL) during empirical therapy died within 17 days, whereas 70% of patients with low maximum *Oxa-51* DNA (<2 log copies/mL) survived (*P* = 0.003, log-rank test) (Figure 4A). Moreover, based on the increase or decrease in *Oxa-51* DNA from day 0 to day 3, two groups of patients had different survival curves (*P*<0.001, log-rank test) (Figure 4B). These were further supported by multivariate Cox's proportional hazard analysis (Table 3). Similarly, the survival curves based on a single day measurement of *Oxa-51* DNA on day 2 or day 3 (>3 or <3 log copies/mL) were significantly different (data not shown).

**Table 3:** Analysis of factors predicting mortality of study patients with *A. baumannii* bacteremia by multivariate Cox's proportional hazard model^a^.

Covariate	*P* value	Adjusted hazard ratio	95% Confidence interval
Pitt bacteremia score	0.02	1.29	1.04–1.60
Change of *Oxa-51* DNA, day 0^b^ – day 3(Δ log copies/mL/day)	0.004	1.87	1.22–2.85
Maximum *Oxa-51* DNA, day 0^b^ – day 3(log copies/mL)	0.09	1.52	0.94–2.45

a Test of proportional-hazards assumption: *P* = 0.14>0.05, Groennesby and Borgan GOF test *P* = 0.23>0.05, adjusted generalized R^2^ = 0.405.

b Day 0 was the day on which the first blood sample positive for *A. baumannii* by culture was drawn.

## Discussion

In light of the rapid increase in the antimicrobial resistance of *A. baumannii*, limited choice of antimicrobial agents and multiple complicated risk factors associated with its high mortality, it is important to identify good parameters for evaluating therapeutic responses to new or combination regimens in well controlled clinical studies [3], [4], [9]. In this study, we established an *Oxa-51* quantitative real-time PCR assay to measure *A. baumannii* loads in blood from patients with *A. baumannii* bacteremia. Compared with the survivors, the nonsurvivors had higher maximum *Oxa-51* DNA load and an increased trend from day 0 to day 3; both parameters and the single day measurement of *Oxa-51* DNA on day 3, the day when blood culture reports were available from most clinical laboratories, were independent predictors for mortality by multivariate analysis (Table 2). To our knowledge, this is the first report demonstrating high *A. baumannii* loads in blood were associated with mortality. Moreover, nonsurvivors receiving appropriate antibiotics showed a trend of less increase in *Oxa-51* DNA during the empirical period compared with those receiving inappropriate antibiotics, suggesting that monitoring *Oxa-51* DNA load in blood could provide simple and direct parameters for evaluating therapeutic response of *A. baumannii* bacteremia in future clinical trials.

Several risk factors for mortality of *A. baumannii* bacteremia have been identified by different studies, such as septic shock [11], [14], high Pitt bacteremia score [15], immunosuppressant [11], [15], age, recent surgery, acute respiratory failure, acute renal failure [11], McCabe score, ventilator usage [14], disseminated intravascular coagulation, and inappropriate antibiotics usage [5]. With one exception [5], the majority of these were retrospective studies, which might be potentially biased by retrospective recall. We carried out a prospective study and included all factors studied in the multivariate logistic regression analysis, and three independent factors, maximum *Oxa-51* DNA, its change from day 0 to day 3 and Pitt bacteremia score were identified (Table 2). These findings suggest both bacterial and host factors are important. The possible role of appropriate empirical antibiotic therapy was suggested by the trend of less increase in *Oxa-51* DNA in the subgroup of nonsurvivors who received appropriate antibiotics compared with those receiving inappropriate antibiotics (Figures 3B and 3C). Analysis of this subgroup and the survivors receiving appropriate empirical antibiotics, of which both had an indistinguishable trend of *Oxa-51* DNA change (Figure 3C), revealed that SOFA score and platelet counts were different (*P* = 0.06 and 0.03, respectively, Mann-Whitney U test, two tailed), suggesting host factors play a key role in determining mortality in this subgroup. The importance of host factors was further suggested by comparing the left panels of Figures 3B and 3C, in which appropriate empirical antibiotics were strongly associated with decreasing *Oxa-51* DNA load (*P*<0.001, Mann-Whitney U test, two tailed), however, all patients in both groups died. This might be due to host factors; most of our patients had high APACHE II score (interquartile range [IQR], 20.5–36), Charlson score (IQR, 2–6) and Pitt bacteremia score (IQR, 3–7) (Tables 1 and 2). Another bacterial factor, antimicrobial resistance, was also carefully examined. Although ERAB was significantly associated with mortality in the univariate analysis, it was not significant in the multivariate analysis (OR, 1.99; 95% CI, 0.21–18.71; *P* = 0.55) (Table 2). Similarly, sulbactam resistance was not significantly associated with mortality in the multivariate analysis (Table 2).

Although SOFA score was strongly associated with mortality in the univariate analysis (Table 2), it was not included in the multivariate analysis due to small case number with SOFA score on day 0 available in our prospective observational study design. Nonetheless, including SOFA score in the multivariate logistic regression analysis revealed a similar final model, which included SOFA (OR, 1.65; 95% CI, 1.02–2.66; *P* = 0.04), change of *Oxa-51* DNA, day 0 – day 3 (OR, 6.93; 95% CI, 1.00–48.15; *P* = 0.05) and maximum *Oxa-51* DNA, day 0 – day 3 (OR, 14.93; 95% CI, 0.65–341.06; *P* = 0.09). The sensitivity, specificity, positive predictive value and negative predictive value were 100%, 87.5%, 95.2%, and 100%, respectively. The area under ROC curve was 0.9688. In multivariate Cox's proportional hazard model analysis, SOFA (Hazard ratio [HR], 1.17; 95% CI, 1.03–1.32; *P* = 0.01) and change of *Oxa-51* DNA, day 0 – day 3 (HR, 1.63; 95% CI, 1.08–2.49; *P* = 0.02) were independent factors for mortality.

Of the 43 follow-up blood cultures from the 51 patients, only 3 (6.98%) were positive and 40 were negative, of which 12 had detectable *Oxa-51* DNA (median 2.0 log/copies/mL, interquartitle range 1.6 to 2.6 log/copies/mL). This could be due to a drastic decrease in the sensitivity of blood culture after antibiotic usage as reported previously [2], [18]. Alternatively, the possibility that the *Oxa-51* DNA detected in these samples was derived from dead or degraded bacteria rather than viable bacteria can not be completely ruled out. A recent study examining the amounts of *Neisseria meningitidis* in blood by quantitative PCR and simultaneous culture reported that the DNA copy numbers were more than 2 logs higher than CFU numbers, suggesting significant amounts of non-viable bacteria were present in vivo [22]. Nonetheless, the good correlation between bacterial DNA load and disease severity and/or outcome demonstrated in different bacterial infections, including *Streptococcus pneumoniae, Neisseria meningitides,* MRSA and *A. baumanii* in this study, strongly suggested bacterial DNA load, though derived from both viable and dead bacteria, could be a useful marker to predict disease outcome [19]–[22].

There are several limitations of this study. First, the sample size was small. Future study with a larger sample size is needed to verify our observations and the applicability of this method in clinical practice. Second, this study enrolled only critically ill patients in ICU. Future study enrolling patients from general wards is also needed to investigate the roles of *Oxa-51* DNA load in non-critically ill patients. Moreover, the relationship of the kinetics of *Oxa-51* DNA load in blood to antibiotic regimens and other factors during the course of infection require further investigation. Nonetheless, our findings that high and increased *Oxa-51* DNA load can predict mortality in *A. baumannii* bacteremia support the notion that measuring bacterial DNA load could predict disease outcome or monitor therapeutic responses, and may have implications to our understanding of pathogenesis, particularly for those bacterial diseases with high mortality, multiple and complicated risk factors.

## Supporting Information

Text S1 Copy of the approval of the Institutional Review Board
(5.08 MB PDF)

Text S2 Study protocol
(0.02 MB PDF)

Text S3 Quantitative real-time PCR assay for Oxa-51 gene of A. baumannii
(0.08 MB DOC)

Text S4 Multivariate logistic regression analysis
(0.04 MB DOC)

Table S1 Antimicrobial susceptibility profile and antibiotic therapy of each study patient with A. baumannii bacteremia.
(0.09 MB DOC)
