# Supplementary material for: High and Increasing Oxa-51 DNA Load Predict Mortality in Acinetobacter baumannii Bacteremia: Implication for Pathogenesis and Evaluation of Therapy
Source: PLoS One. 2010 Nov 30;5(11):e14133. doi: 10.1371/journal.pone.0014133 (PMC2994729; doi:10.1371/journal.pone.0014133)
Supplement: Text S1 — Copy of the approval of the Institutional Review Board (5.08 MB PDF) [file pone.0014133.s001.pdf]

Approval of the Institutional Review Board of National Taiwan University Hospital

檔　號：
發文方式：郵寄
保存年限：

國立臺灣大學醫學院附設醫院　函

地址：100臺北市中山南路7號
承辦人：戴君芳
電話：02-2312-3456轉63160
傳真：02-2395-1950
電子信箱：NTUHREC2008@YAHOO.COM.TW

受文者：內科部莊祐中醫師
發文日期：中華民國97年10月7日
發文字號：校附醫倫字第0971761064號
速別：普通件
密等及解密條件或保密期限：普通
附件：

主旨：有關　台端所主持之「利用聚合酶連鎖反應定量鮑氏不動桿菌血液中菌量/ Quantification of Acinetobacter baumannii in blood by a real-time polymerase chain reaction assay」（本院案號：200809045R）純學術臨床試驗案，符合快速審查條件及研究倫理規範，通過本院研究倫理委員會審查，同意核備，並提第144次研究倫理委員會報備追認，請　查照。

說明：

一、為配合WHO之稽核及符合ICH 4.10.1之規範「The Investigator should written summaries of the trial status to the IRB/IEC annually, or more frequently, if requested by the IRB/IEC.」，凡通過本會審查之案件，需於追蹤年限1年到期前的1個月內提出期中報告及持續追蹤審查表，逾期未繳交者，不得申請新案。

二、依據國際醫學雜誌編輯委員會(The International Committee of Medical Journal Editors, ICMJE)之投稿規定(http://www.icmje.org/clin_trialup.htm)，臨床試驗研究計畫投稿者，需於招募第一位受試者參與試驗前，將通過研究倫理委員會審核之臨床試驗計畫資料登錄於臨床試驗公開網站，完成登錄作業後，國際醫學雜誌編輯委員會(

ICMJE)才會接受研究結果之發表。WHO對臨床試驗研究計畫之定義為任何對受試者或特定族群進行一個或多個與健康有關的介入措施(如藥物、外科處置、器材、行為治療、飲食介入及照護過程改變)以評估對健康的效益之計畫，非屬上述臨床試驗計畫，請計畫主持人自行決定是否登錄。

三、本院已向美國國家衛生研究院(National Institutes of Health, NIH) ClinicalTrials.gov 網站- Protocol Registration System (PRS https://register.clinicaltrials.gov/)申請本院專用帳號，供本院計畫主持人(PI)登錄所主持之臨床試驗研究計畫，登入網頁之帳號及密碼如下列：

(一)Organization：[redacted]

(二)User Name [redacted]

(三)Password：[redacted]

正本：內科部莊祐中醫師

副本：研究倫理委員會

院長 陳明豐

台大醫院研究倫理委員會

申請<u>其他類</u>臨床試驗計畫送件核對單

（本清單請置於首頁）

計畫名稱: 利用聚合酶連鎖反應定量鮑氏不動桿菌血液中菌量

申請人科部/姓名：內科部感染科 莊祐中醫師

請依下列表單順序置放，並勾選您已檢附之申請表格：

項次	表單	備齊(V)	備註	REC 收件人確認欄(V)
1	台大醫院研究倫理委員會臨床試驗計畫申請書	V	申請人與科/部主任需簽章	
2	臨床試驗計畫書	V	申請人需於首頁簽章	
3	台大醫院研究倫理委員會臨床試驗快速審查範圍評檢表	V	若符合者	
4	台大醫院研究倫理委員會臨床試驗受試者說明及同意書	無	申請人需於首頁簽章	
5	問卷	無	若有	
6	招募受試者廣告文宣品	無	若有	
7	個案報告表(Case Report Form)	無	若有	
8	主持人手冊（Investigator's Brochure）	無	若有	
9	參考文獻	無		
10	主持人及協同研究人員之學經歷、著作及所受訓練之背景資料	V	申請人需於首頁簽章	
11	電子檔案 1 份：內含上述所附申請文件、臨床試驗許可書	V	可用隨身碟當場存取，或 e-mail 至倫委會(e-mail: ntuhreo11@yahoo.com.tw)	

上述申請文件請以資料夾裝訂並依序置放表單、加註標示，並準備 1 式 2 份；送件地點為台北市中山南路 7 號台大醫院東址北棟地下 4 樓研究倫理委員會。

送件人簽章/日期：

倫委會填寫收件人簽章/日期：________________

☐ 文件不足，請補件

☐ 確認送件資料無誤

Application to the Institutional Review Board
of National Taiwan University Hospital.

台大醫院研究倫理委員會

臨床試驗計畫申請書

計畫編號				
計畫名稱	中文：利用聚合酶連鎖反應定量鮑氏不動桿菌血液中菌量			
	英文：Quantification of Acinetobacter baumannii blood by a real-time polymerase chain reaction assay			
計畫主持人	(中/英文姓名) 莊祐中 Yu-Chung Chuang			
	服務單位	內科部感染科	職稱	醫師
	聯絡電話	9807843	電子郵件	yuchung@ntuh.gov.tw
協同主持人	姓名			
	服務單位		職稱	
	聯絡電話		電子郵件	

臨床試驗基本資料

1.使用的藥物、醫療器材或技術名稱：	利用同步定量聚合連鎖反應偵測病人鮑氏不動桿菌菌血症
2.研究計畫目的：	鮑氏不動桿菌為革蘭氏陰性桿菌，它可以在生物及非生物上生存，或是在人類身上群聚。它甚至可以在乾燥的非生物表面生存高達數天的時間，目前是越來越重要的伺機性致病菌,尤其對於重症的病患造成伺機性的感染更是重要。<br>鮑氏不動桿菌近年來漸成為重要的院內致病菌，尤其是加護病房的病患，免疫低下的病患，或是燒燙傷的病患。由於其特殊的生物特性，天然就對數種抗生素俱有抗藥性，且對其他抗生素極容易產生抗藥性，漸漸造成嚴重的多重抗藥性菌株院內感染。也因此對於鮑氏不動桿菌能選擇的抗生素也就越來越受到限制。感染部位以呼吸道感染最常見，其次為血流感染、泌尿道感染，皮膚及軟組織感染等。多重抗藥性的鮑氏不動桿菌所造成的肺炎及菌血症，其死亡率相當高，國外研究發現歸因於多重抗藥性菌株感染的死亡率高達 25%，而台大自己的研究則是三十天的死亡率將近 50%。<br>部份鮑氏不動桿菌所造成菌血症所造成的死亡在發病後一兩天就發生，是否因血中菌量過高而導致早期死亡並沒有過相關研究。所以本研究計畫希望能透過同步定量聚合連鎖反應偵測病人鮑氏不動桿菌菌血症，希望建立起菌量及預後的關係
3.預期試驗期限：	即日起至 2009/06/30
4.受試驗者數目：	40

5. 請勾選試驗類別：（可複選）

☐ 藥品類(請加填第 7、8 項)

☐ 新醫療器材或技術類：☐第一級 ☐第二級 ☐第三級

V 其他

6. V 符合快速審查條件(請加填快速審查範圍評檢表)

☐不符合快速審查條件

7. 藥品臨床試驗類別：（可複選）不適用

☐ 無衛生署藥品許可證，為申請查驗登記

☐ 無衛生署藥品許可證，為純學術研究

☐ 多國多中心藥品臨床試驗

☐ 已有衛生署許可證，為新增適應症

☐ 已有衛生署許可證，為純學術研究（請加勾：☐非本院進用藥品；☐本院進用藥品）

☐ 無衛生署藥品許可證，為做BA、BE、PK、PD臨床試驗

8. 試驗藥品基本資料：不適用

學名： 商品名： 含量：

製造廠商，國別：

藥物發展階段：☐Phase I ☐Phase II ☐Phase III ☐Phase IV ☐其他________

9. 研究計畫責任歸屬：

☐贊助者: 公司／機構：________________

☐台大醫院 V 研究主持人 ☐其它:______________________________

10. 研究計畫之資金贊助來源（可複選）：

☐藥品／設備製造商：__________________

☐衛生署 ☐國科會 ☐國家衛生研究院 ☐台大醫院

V 大學或科部__________________________ ☐其他：______________________________

11. 使用藥物或器材提供者（可複選）：

☐廠商：___________________

☐其他學術單位：___________

V 常規醫療處置

12. 研究計畫是否包括下列參與人員（可複選）：

V 生物統計專家

☐研究資料處理員

☐研究資料稽查單位

☐安全監督委員會

☐不適用

13.研究計畫設計種類：

☐隨機單盲

☐隨機雙盲

☐本國多中心，協調中心，名稱：______________

☐多國多中心

V 其他：Retrospective study

14.受試者資料：

(1) 每位受試者預期之試驗期限：回溯性試驗

(2) 受試者型態（如：對照組之健康人，罹患特殊疾病之病患）：加護病房內因鮑氏不動桿菌菌血症早期死亡的病患

(3) 於本院受試之估計人數(人)：40，其他單位人數(人)：0

(4) 年齡範圍：20歲以上

(5) 是否有依性別選擇受試者？ ☐是：☐男性☐女性；　V 否

(6) 如受試者有懷孕之可能，此研究是否包括懷孕檢測？

☐是　V 否，請說明：抽血檢查，不影響懷孕過程

(7) 如受試者為孩童或殘障人士，請說明需要此類受試者之原因：殘障人士也會因鮑氏不動桿菌菌血症死亡

(8) 請說明主持人與受試者之關係？

☐老師／學生　V 研究者／受試者　☐醫療人員／病人　☐雇主／職員

☐朋友　☐其它，請說明：________________

15. V 申請免除受試者同意書，原因：研究用人體檢體採集與使用注意事項十四點第二項：因無法追蹤或聯絡等原因，難以重新取得檢體提供者同意

病人因菌血症發病時就會定期追蹤全血，而本研究只回溯性分析病人的臨床資料及檢驗結果，以及回溯性使用這些病人已抽血的剩餘檢體，做細菌量的分析，不牽涉病人本身的分析，也不影響病人權益。而且因為本研究在研究早期死亡的病人，病人因為菌血症而導致早期死亡，在細菌檢驗室報告時，病人都以經死亡且離院，難以再取得檢體提供者同意。但因鮑氏不動桿菌菌血症會造成很高的死亡率，值得做進一步的分析。

臨床試驗計畫申請人自評表

（為提昇您在撰寫[illegible]及提昇審查速率，請仔細閱讀下列注意事項，並勾選您所撰寫之內容已涵蓋者，請[illegible]項不適用者，請於最右欄位，說明原因。）

15.The informed consent is exempt for the following reasons.
The blood samples for this study are aliquots from the patients' routine blood sampling during their stay in the ICU, and there will be no additional blood drawing for this study. The treatment and health care of the patients during the study period will neither be affected by the enrollment status of the patients nor by the data derived from this study. All the patients enrolled and all the blood samples collected will be "coded". All the data will be analyzed anonymously.

V	☐	試驗目的及背景說明（包括學理根據及有關文獻報告）

V	☐	試驗方法與程序
V	☐	受試者選擇標準
V	☐	試驗設計與流程（與臨床試驗相關部份）
V	☐	試驗期限與進度
V	☐	所需藥品或醫療器材名稱及數量
V	☐	資料之蒐集處理評估及統計分析方法
V	☐	臨床上不良反應及處理方法
V	☐	前臨床試驗參考資料
V	☐	如尚屬研究中之新藥或新醫療器材，應說明其現況，並檢附生產國及其他國家核准進行臨床試驗之證明文件影印本
V	☐	身心上可能產生之危險性與獲得之利益
V	☐	財務上可能產生之危險與獲得之利益
V	☐	利益衝突
V	☐	其他資料/文獻
2. 若屬新醫療技術/器材類案件，計畫書內容附加：不適用		
符合	不適用	內容
☐	V	新醫療器材之圖樣及說明書。
☐	V	出產國最高衛生機構出具之製造證明或核准施行臨床使用及臨床試驗之證明影本，其內容包括製造廠名稱、廠址、器材名稱、規格、型態(或效能)、製造情形，以及准其在出產國測試或販賣實況(歸類第三級者免，但需提出實驗室、動物實驗研究報告)。
☐	V	臨床試驗報告書(但歸類第三級者免)。
☐	V	效果理論報告書。
☐	V	屬電氣類之新醫療器材者，應具電氣配置圖、醫療儀器電性安全測試報告以及裝置之使用手冊。
☐	V	屬可發生游離輻射設備或使用放射性物質者，應具符合原子能法有關規定之證明文件影本。
☐	V	其他所應遵循之安全及性能品質標準資料。
**II. 臨床試驗受試者說明及同意書檢核表：**		
通則		
符合	不適用	內容
☐	V	受試者說明及同意書全文請以「標楷體」字型撰寫，各節字體大小級數請依照下列規定繕打：段落標題：14 點、粗體，段落間距：1.5 倍行高，內文：12 點、固定行高 20pt。
☐	V	受試者說明及同意書內容請以通順、口語化之中文詳述，避免直接翻譯，應注意語意通順及避免錯字產生，應以國三程度者能夠理解為原則。
☐	V	請加註 24 小時聯絡人的姓名及手機號碼。

□	V	受試者說明及同意書提及「受試者」或「病患」處，請統一以第二人稱(您)取代，若為小兒科用藥試驗，可提及您的小孩。
一、 藥品(新醫療技術、新醫療器材或其他)全球上市現況簡介：不適用		
符合	不適用	內容
□	V	請說明本試驗藥物/器材是否有衛生署藥品許可證或其他國家許可證。
□	V	請註明本試驗用藥(或產品)目前的研發狀況。
二、 試驗目的：		
符合	不適用	內容
□	V	請以口語化之中文敘述試驗用藥(或產品)與試驗主題之間關聯性。
三、 試驗之主要納入與排除條件：		
符合	不適用	內容
□	V	受試者說明及同意書提及「納入條件」或「排除條件」處，請統一以「符合下列條件者，適合參加本試驗」及「若有下列情況者，不能參加本試驗」等明白易懂的字句取代。
□	V	請註明本院及其他國家及醫院之預定收納人數。
四、 試驗方法、程序及相關檢驗：		
符合	不適用	內容
□	V	請以口語化之中文詳述試驗方法與程序。
□	V	請註明本試驗用藥(或產品)之使用時間、劑量、過程、用法。
□	V	請以淺顯易懂之文字說明「...開放、配對式、隨機比較...」等名詞。
□	V	請註明對照組使用藥品(或產品)之藥效及相關訊息。
□	V	請於受試者說明及同意書中增列特殊檢查之施行方式及危險性。
□	V	請註明試驗期間返診之頻率、各項檢查項目、剩餘藥品(產品)處理方式及評估工具。
□	V	請以中英文並列方式說明檢測項目；因國情不同，提及度量單位時，請統一以「c.c.」取代。
五、 可能產生之副作用、危險或不適之發生率及處理方法：		
符合	不適用	內容
□	V	請註明可能產生的不良反應或副作用之發生比率。
□	V	對於可能發生之不良反應或副作用，是否有追踪及檢驗方式。
□	V	請註明受試者於何種狀況下應停藥。
□	V	請註明對胚胎、胎兒、嬰兒授乳的可能危險性及處理方式。
□	V	為保護病人權益，請將容易造成受試者危險或警示字句以粗黑體字標示。
□	V	若試驗婦女在參加試驗期間不得懷孕，請以粗黑字體標示。

☐	V	請教導並提醒受試者：在試驗期間如發生嚴重疾病或意外事件時，若無法立即聯絡到計劃相關人員，儘可能先至本院急診室就醫，並主動告知醫護人員有加入臨床試驗，亦可請本院總機同仁利用 PHS 協尋計劃主持醫師或協同主持醫師(或製作小卡片讓受試者隨身攜帶，卡片上可載明計畫主持醫師姓名、計畫名稱、用藥情況、受試者家屬緊急聯絡方式及計畫相關人員姓名及聯絡方式....等資訊)。
六、 本疾病相關之其他可能替代療法及療程說明：		
符合	不適用	內容
☐	V	儘可能說明現行各類療法之利弊。
☐	V	請將醫師專用術語口語化。
七、 試驗預期效益：		
符合	不適用	內容
☐	V	請註明試驗藥物(或產品)於國內外試驗之結果。
☐	V	不可誇大試驗療效。
八、 試驗進行中受試者之禁忌、限制與應配合事項：		
符合	不適用	內容
☐	V	請註明禁止的合併用藥、飲食及日常活動之項目。
九、 機密性		
符合	不適用	內容
☐	V	請註明贊助者名稱。
十、 相關損害發生時的賠償、治療與保險：		
符合	不適用	內容
☐	V	由廠商贊助之臨床試驗案件請用第 1 項，由本院醫師自行發起之臨床試驗案件請用第 2 項，並請自行刪除不需要之項目。
☐	V	若對本臨床試驗或受試者有提供保險或其他相關資訊，請加註說明。
十一、受試者權利：		
符合	不適用	內容
☐	V	臨床試驗期間之試驗用藥、各項檢查費用之支付方式。
☐	V	額外的報酬，例如車馬費、營養費……等。
☐	V	請加註試驗主持人之姓名及聯絡電話。
☐	V	有新資訊會隨時通知受試者。
十二、試驗之退出與中止：		
符合	不適用	內容
☐	V	讓受試者明白參與此研究是自願性的，因此可以在任何時間退出，而不會有任何歧視、處罰或權益喪失的問題。
十三、利益衝突：		
符合	不適用	內容

□	V	如無利益衝突，請註明〞無〞。
十四、簽章：		
符合	不適用	內容
□	V	1式2份，簽署完畢後，正本由試驗主持醫師留存，副本由簽署人留存。
**III. 科學與倫理審查重點**		
1.計畫設計與執行：		
符合	不適用	內容
V	□	於本院執行之適當性，包括其醫事人員、設施、及處理緊急狀況之能力。
V	□	計畫主持人的資格及經驗之適當性。
V	□	試驗設計與目的之合理關聯性、統計方法（包括樣本數計算）之合理性及依最低
V	□	受試者人數達成妥適結論之可能性。
V	□	預期風險與預期效益相較之合理性。
V	□	選擇對照組之合理性
V	□	受試者提前退出試驗之條件。
V	□	暫停或中止全部試驗的條件。
V	□	監測與稽核試驗進行之規定是否充足；是否組成資料安全監測委員會。
V	□	試驗結果之報告或發表方式。
2. 潛在受試者之招募：		
符合	不適用	內容
V	□	潛在受試者所存在之母群體特性（包括性別、年齡、教育程度、文化、經濟狀態及種族淵源）。
V	□	最初接觸與召募進行之方式。
V	□	將全部資訊傳達予潛在受試者之方式。
V	□	受試者納入條件
V	□	受試者排除條件
3. 受試者之照護：		
符合	不適用	內容
□	V	對受試者心理及社會層面之支持。
□	V	為試驗目的而取銷或暫停標準治療之合理性。
□	V	試驗期間及試驗後，提供受試者之醫療照護。
□	V	試驗過程中，受試者自願退出時，將採取之步驟。
□	V	試驗產品延長使用、緊急使用及恩慈使用之標準。
□	V	於受試者同意下，通知受試者家庭醫師之程序。
□	V	計畫結束後，提供受試者繼續取得試驗產品之計畫。
□	V	參加試驗對受試者財務狀況之可能影響。
□	V	受試者之補助及補償。

☐	V	受試者因參與試驗而受傷、殘障或死亡時之補償與治療。
☐	V	賠償及保險之安排。
4. 受試者隱私之保護：		
符合	不適用	內容
V	☐	記載可能接觸受試者個人資料（包括其醫療記錄及檢體）之人。
V	☐	為確保受試者隱私和個人資訊安全所採之措施。
5. 受試者同意：		
符合	不適用	內容
☐	V	取得受試者同意之相關程序。
☐	V	提供受試者或其合法代理人完備之書面或口頭資料。
☐	V	將不能行使同意者納入試驗之理由。
☐	V	於試驗期間，確保受試者及時得到與其權利、安全與福祉相關之最新資訊。
☐	V	於試驗期間，接受受試者或其代理人之詢問或投訴並予以回應之機制。
6. 其他：		
符合	不適用	內容
☐	V	研究對受試者所屬社群或社區將造成何種影響與關聯？
☐	V	在研究設計上，對相關社群採取了哪些諮詢步驟？
☐	V	社群對其成員在進行同意時是否產生什麼影響？
☐	V	審查所提之研究過程中採用之社群諮詢方式。
☐	V	研究對於社群本身能力的建立會有何種程度的貢獻？
☐	V	是否說明有關受試社群在試驗成功後可取得、能支付研究之產品？

若於上述自評表中有勾選任一項不適用者，請統一說明原因於此欄位：

原因說明：本研究無治療其它介入性研究，且是回溯性的分析

本案連絡人：莊祐中	
服務單位：內科部感染科	職稱：醫師
聯絡電話：0968457843	電子郵件：yuchung@ntuh.gov.tw
地址：台大醫院內科部感染科	
計畫申請人簽章/日期	科/部主任簽章/日期

國立臺灣大學醫學院附設醫院
臨床試驗計畫書

*本試驗計劃書應在首頁附加「臨床試驗內容摘要表」

□藥品 □醫療器材 □醫療技術 ☑其他 血液採檢

一、計畫名稱：

中文：利用聚合酶連鎖反應定量鮑氏不動桿菌血液中菌量

英文：Quantification of *Acinetobacter baumannii* in blood by a real-time polymerase chain reaction assay

二、主要主持人：莊祐中醫師

三、協同主持人：

四、試驗主題：利用聚合酶連鎖反應定量鮑氏不動桿菌血液中菌量

五、試驗目的及背景說明（包括學理根據及有關文獻報告）：

鮑氏不動桿菌為革蘭氏陰性桿菌，它可以在生物及非生物上生存，或是在人類身上群聚。它甚至可以在乾燥的非生物表面生存高達數天的時間，目前是越來越重要的伺機性致病菌,尤其對於重症的病患造成伺機性的感染更是重要[1]。

鮑氏不動桿菌近年來漸成為重要的院內致病菌，尤其是加護病房的病患[2]，免疫低下的病患[3]，或是燒燙傷的病患[2]。由於其特殊的生物特性，天然就對數種抗生素俱有抗藥性，且對其他抗生素極容易產生抗藥性，漸漸造成嚴重的多重抗藥性菌株院內感染[4]。也因此對於鮑氏不動桿菌能選擇的抗生素也就越來越受到限制。感染部位以呼吸道感染最常見[5]，其次為血流感染、泌尿道感染，皮膚及軟組織感染等[6]。多重抗藥性的鮑氏不動桿菌所造成的肺炎及菌血症，其死亡率相當高，國外研究發現歸因於多重抗藥性菌株感染的死亡率高達 25%[7]，而台大自己的研究則是三十天的死亡率將近 50%。[8]

過去對於預後因子的研究主要著重在是不是某些臨床指標，或是臨床用藥對於預後有其關聯性,而就台大自己之前所做的研究則是指出預後只跟病人的原本的疾病嚴重度及病人是否有免疫抑制的狀況有關[8]，但是在過去的這些研究中並沒有對於菌量的探討。

近年來，菌血症的診斷工具日益精進[9]，某些 GNB 或 GPC 針對以 PCR 作為快速診斷，已有商業用的組套(kit)可供使用[10]。在 Jaffe 等人[11]發展出從血液培養瓶中以 polymerase chain reaction (PCR)偵測得細菌的 DNA 後，Louie 等人[12]進一步證實以 16S rRNA, *mecA, nuc* 等引子能得到 99%以上的敏感度及特異性。

雖然由培養瓶中測得細茵的 DNA 或 RNA 的技術已臻成熟，由於每次打入培養瓶的血量不同，因此不適合以培養瓶中的 DNA 或 RNA 含量作為定量依據。針對血液檢體直接測 PCR 甚至於定量的研究仍相當有限。Heininger[13]等人測試了全血中 16S ribosomal RNA 基因作為菌血症探測法,測試了包括革蘭氏陽性的金黃色葡萄球菌及革蘭氏陰性的大腸桿菌的 sensitivity 及 specificity。Such[14] 針對二十八位肝硬化，腹水培養為陰性的病患，利用 16S ribosomal RNA 基因，從其中九位病患的血清及腹水中驗得致病的細茵。此一研究團隊[15]更進一步利用此一 16S ribosomal RNA 基因及 real-time PCR，在病人住院時及每八小時後檢驗 DNA 量(relative quantification)，用以推估細菌清除的(bacterial clearance)時間。

以 DNA 作為定量方式是否正確曾有爭議。Ludwig[16]曾指出，由於細菌在不同生長期 DNA copy number 不同，因此 DNA copy number 不一定能代表細菌數。因此 Sabersheikh[17]試圖以探測 RNA 的量作為定量方式：取得 MRSA 菌株的 RNA 後，以 random priming 反轉錄成 complementary DNA (cDNA)；以 10^4-10^9 copies/μL 16S rRNA 為 internal control，就可以定量 RNAIII，*hla* (alpha haemolysin) 及 *spa* (protein A)。在此研究中，16S rRNA 在不同生長期的量是恒定的，因此可作為 internal control，而 Eleaume[18]也有相似的結果。

在 *Staphylococcus epidermidis* 上，Vandecasteele 等人[19]曾以 quantitative PCR 在動物實驗中以 genomic DNA 及 RNA 在靜脈導管的量作為療效的評估依據，兩者有很好的相關性。不過在 *Acinetobacter baumannii* 方面，和在血液檢體上兩者的相關性研究則還沒有。Turton[20]等人以 PCR 的方式証實 *Acinetobacter baumannii 特異的 blaOXA-51-like carbapenemase gene*。而 Hu[21]等人也有本土的資料証實本土的菌株也確實帶有 *blaOXA-51-like carbapenemase gene*。

部份鮑氏不動桿菌所造成菌血症所造成的死亡在發病後一兩天就發生，是否因血中菌量過高而導致早期死亡並沒有過相關研究。所以本研究計畫希望能透過同步定量聚合連鎖反應偵測病人鮑氏不動桿菌菌血症，希望建立起菌量及預後的關係

六、試驗方法：

(一)受試者選擇標準 (Patient eligibility)

住院病患，因 *Acinetobacter baumannii* 血液培養陽性，菌血症而早期死亡者

(二)試驗設計與流程 (與臨床試驗相關部份)：

血液培養由採檢到陽性反應約需三至五日。由於病患於臨床上懷疑菌血症做血液培養時，皆會同時收集全血球計數(CBC)及生化檢體，檢體會在檢驗室保存一週以上，檢體收集較為簡易可行。當血液培養報告為 *Acinetobacter baumannii* 陽性時，若病人早期因敗血症死亡，回溯使用之前所保存在檢驗室的血液檢體，檢驗當中細菌的量。

(三)試驗期限與進度

預計進行一年, 即日至民國九十八年六月三十日

(四)所需藥品或醫療器材名稱及數量: 無

(五)病歷記錄用紙之格式: 不需特殊記載

(六)資料之蒐集處理評估及統計分析方法:

取得檢體後, 以定量 PCR 檢測血中菌量, 檢驗每位病人的菌量, 並同時記錄病人的臨床症狀及其它臨床檢驗結果做為分析。

七、臨床上不良反應及處理方法：因為回溯性分析使用剩餘檢體, 對健康情形並無不良反應.

八、主持人及協同研究人員之學經歷、著作及所受訓練之背景資料：

主持人: 莊祐中醫師

台大醫學系畢業, 台大醫學院臨床醫學研究所碩士班肄業

台大愛滋病防治中心專案主治醫師

九、受試者說明及同意書格式：無

十、前臨床試驗參考資料：

1. Manikal, V.M., et al., *Endemic carbapenem-resistant Acinetobacter species in Brooklyn, New*

York: citywide prevalence, interinstitutional spread, and relation to antibiotic usage. Clin Infect Dis, 2000. **31**(1): p. 101-6.
2. Cisneros, J.M., et al., *Bacteremia due to Acinetobacter baumannii: epidemiology, clinical findings, and prognostic features.* Clin Infect Dis, 1996. **22**(6): p. 1026-32.
3. Levin, A.S., et al., *Severe nosocomial infections with imipenem-resistant Acinetobacter baumannii treated with ampicillin/sulbactam.* Int J Antimicrob Agents, 2003. **21**(1): p. 58-62.
4. Cisneros, J.M. and J. Rodriguez-Bano, *Nosocomial bacteremia due to Acinetobacter baumannii: epidemiology, clinical features and treatment.* Clin Microbiol Infect, 2002. **8**(11): p. 687-93.
5. Baraibar, J., et al., *Risk factors for infection by Acinetobacter baumannii in intubated patients with nosocomial pneumonia.* Chest, 1997. **112**(4): p. 1050-4.
6. Rodriguez-Bano, J., et al., *Clinical features and epidemiology of Acinetobacter baumannii colonization and infection in Spanish hospitals.* Infect Control Hosp Epidemiol, 2004. **25**(10): p. 819-24.
7. Lortholary, O., et al., *Nosocomial acquisition of multiresistant Acinetobacter baumannii: risk factors and prognosis.* Clin Infect Dis, 1995. **20**(4): p. 790-6.
8. Tseng, Y.C., et al., *Prognosis of adult patients with bacteremia caused by extensively resistant Acinetobacter baumannii.* Diagn Microbiol Infect Dis, 2007.
9. Smyth, R.W., et al., *Methods for identifying methicillin resistancein Staphylococcus aureus.* J Hosp Infect, 2001. **48**(2): p. 103-7.
10. Wellinghausen, N., et al., *Evaluation of the Hyplex BloodScreen Multiplex PCR-Enzyme-linked immunosorbent assay system for direct identification of gram-positive cocci and gram-negative bacilli from positive blood cultures.* J Clin Microbiol, 2004. **42**(7): p. 3147-52.
11. Jaffe, R.I., et al., *Rapid extraction from and direct identification in clinical samples of methicillin-resistant staphylococci using the PCR.* J Clin Microbiol, 2000. **38**(9): p. 3407-12.
12. Louie, L., et al., *Rapid detection of methicillin-resistant staphylococci from blood culture bottles by using a multiplex PCR assay.* J Clin Microbiol, 2002. **40**(8): p. 2786-90.
13. Heininger, A., et al., *Effect of comprehensive validation of the template isolation procedure on the reliability of bacteraemia detection by a 16S rRNA gene PCR.* Clin Microbiol Infect, 2004. **10**(5): p. 452-8.
14. Such, J., et al., *Detection and identification of bacterial DNA in patients with cirrhosis and culture-negative, nonneutrocytic ascites.* Hepatology, 2002. **36**(1): p. 135-41.
15. Frances, R., et al., *A sequential study of serum bacterial DNA in patients with advanced cirrhosis and ascites.* Hepatology, 2004. **39**(2): p. 484-91.
16. Ludwig, W. and K.H. Schleifer, *How quantitative is quantitative PCR with respect to cell counts?* Syst Appl Microbiol, 2000. **23**(4): p. 556-62.
17. Sabersheikh, S. and N.A. Saunders, *Quantification of virulence-associated gene transcripts in epidemic methicillin resistant Staphylococcus aureus by real-time PCR.* Mol Cell Probes, 2004. **18**(1): p. 23-31.
18. Eleaume, H. and S. Jabbouri, *Comparison of two standardisation methods in real-time quantitative RT-PCR to follow Staphylococcus aureus genes expression during in vitro growth.* J Microbiol Methods, 2004. **59**(3): p. 363-70.
19. Vandecasteele, S.J., et al., *The effect of systemic antibiotics on the microbiological diagnosis of experimental foreign body infections caused by Staphylococcus epidermidis.* Diagn Microbiol Infect Dis, 2004. **48**(2): p. 89-95.
20. Turton, J.F., et al., *Identification of Acinetobacter baumannii by detection of the blaOXA-51-like carbapenemase gene intrinsic to this species.* J Clin Microbiol, 2006. **44**(8): p. 2974-6.
21. Hu, W.S., et al., *An OXA-66/OXA-51-like carbapenemase and possibly an efflux pump are associated with resistance to imipenem in Acinetobacter baumannii.* Antimicrob Agents Chemother, 2007.

十一、出產國及核准上市國最高衛生主管機關許可製售證明影印本：無

十二、如尚屬研究中之新藥或新醫療器材，應說明其現況，並檢附生產國及其他國家核准進行臨床試驗之證明文件影印本：無

十三、身心上可能產生之危險性與獲得之利益：因為回溯性收血使用剩餘檢體，對健康情形並無不

良反應

十四、財務上可能產生之危險與獲得之利益：由研究經費支付研究用檢驗項目，無須病人自費或健保額外給付.

十五、利益衝突：無

十六、其他資料：無

台大醫院研究倫理委員會臨床試驗快速審查範圍評檢表

(一) 請自行勾選符合本院規定之快速審查條件:

**V 新案** (若為新案，請勾選符合快速審查之條件 A~E)
**A. 有關藥品臨床研究：**<br>☐ 試驗藥品已有衛生署藥品許可證且為本院藥委會通過使用之藥品，試驗性質為學術研究，且依標準建議劑量及用法用於衛生署已許可適應症，且未與其他藥品併用。<br>☐ 學名藥之單純藥動學試驗。
**B. 從手指、腳跟、耳朵採血或靜脈穿刺收集血液檢體：**<br>☐ 受試者為健康且未懷孕的成年人，體重達 50 公斤以上，八週內採血量不超過 550 毫升，且一週內採血次數不超過二次。<br>☐ 受試者為其他成年人、小孩，需考慮年齡、體重、健康情形、採血步驟、採血量、採血頻率等；八週內採血量不超過 50 毫升或每公斤體重 3 毫升的量，且一週內採血次數不超過二次。
**C. 為研究目的，以前瞻性的非侵入性方法收集生物檢體，例如：**<br>☐ 以不破壞美觀的方式收集頭髮或指甲。<br>☐ 收集剝落的乳齒或因醫療常規所需而拔除的乳齒。<br>☐ 收集醫療常規拔除的永久齒。<br>☐ 收集排泄物和外在分泌物(包括汗水) 。<br>☐ 以不刺激的方式或以咀嚼口香糖、蠟或檸檬酸刺激舌頭後，以非套管方式取得唾液。<br>☐ 收集醫療常規生產時排出的胎盤。<br>☐ 收集醫療常規羊膜穿刺或分娩破水時的羊水。<br>☐ 收集牙齦內牙菌斑及牙結石，需使用有根據的技術方法，且過程中的侵犯性不能超過常規洗牙程序。<br>☐ 由口腔、皮膚刮取或漱口後，取得黏膜和皮膚細胞。<br>☐ 蒸氣吸入後取得痰液。
**D. 以臨床常規使用的非侵入性程序檢查(不包括使用全身麻醉或鎮靜)收集資料，但不包括使用 x-ray、微波。所使用的醫療器材需已獲衛生主管機關核准上市使用。例如：**<br>☐ 使用生理感應器於身體表面或有一段距離之處，但不牽涉到對受試者輸入物質或相當劑量的能量，或是侵犯到其隱私。<br>☐ 量體重、感覺測試。<br>☐ 核磁共振造影。<br>☐ 心電圖、腦波圖、溫度紀錄器、自然界所產生放射性之探測、視網膜電圖、超音波、診斷性紅外線造影、杜普勒血流檢查及心臟超音波檢查。<br>☐ 依受試者年齡、體重和健康情形之適度運動、肌肉強度測驗、體能評估、彎曲性測驗。
E. **其他:**<br>V 研究為例行臨床治療或診斷所收集之資料、文件、記錄、病理標本、菌株、細菌、病毒、生物檢體。<br>☐ 為研究目的而做的錄音、錄影、數位或影像資料記錄。<br>☐ 研究個體或群體特質、行為(包括研究有關知覺感受、認知、動機、認同、語言、溝通、文化信仰或習慣和社會行為等)或研究涉及調查，會談，口述歷史，特定族群，計劃評估，人為因素評估或品質保證的方法。
☐ 不符合以上 A~E，但您認為基於您的研究計畫的某些特殊性質仍符合快速審查的條件。<br>請詳細說明：____
☐ **變更案 (適用於先前已通過本院審查之案件):REC No.:________________**

☐	研究計畫極微的變更，例如：此變更不會有害於危險-利益比例；此變更不會影響已加入之受試者或可能參加本研究的人的參與意願；以及此變更不會影響科學的正當性。
☐	研究計畫已不再收錄新受試者；所有受試者皆已完成所有相關的臨床試驗；受試者仍需長期追蹤。
☐	沒有新受試者的加入，且沒有發現新的危險性。
☐	剩餘的研究僅限於資料分析。

(二)新案快速審查者請回答下列有關危險評估的問題:

是	否	
☐	V	1. 您的研究對象是否為精神疾病患者?
☐	V	2. 您的研究對象是否為藥物濫用者?
☐	V	3. 您的研究對象是否包括胚胎、孕婦或新生兒?
☐	V	4. 您的研究對象是否為監獄受刑人?
☐	V	5. 您的研究對象是否為無法自由表達同意權利的人?
☐	V	6. 您的研究對象是否與研究者有從屬關係，例如：老師/學生或長官/下屬等?
☐	V	7. 您的研究是否涉及精神評估或治療?
☐	V	8. 您的研究是否涉及給予受試者身體上的刺激、誘發疼痛或侵入性處置?
☐	V	9. 您的研究是否涉及給予受試者未經衛生署許可之藥物或未依標準建議劑量及用法用於衛生署已許可適應症?
☐	V	10. 您的研究是否採用隨機分組設計?
☐	V	11. 您的研究是否涉及使受試者接受放射線暴露?
☐	V	12. 您的研究是否涉及基因研究?
☐	V	13. 您的研究是否涉及生殖醫學，例如：不孕症、妊娠終止等?
☐	V	14. 您的研究是否有可能危害研究者，例如：暴露於不安全的環境等?

以上問題，如果您的回答皆為”**否**”，則**符合**進入快速審查流程；

如果您的回答中有任一個”**是**”，請詳加說明您認為符合快速審查的理由:

申請單位主管：單位______________ 簽章___________

申　請　者：單位______________ 簽章___________

國立台灣大學醫學院附設醫院

主持人及協同研究人員之學經歷、著作及所受訓練之背景資料

主持人: 莊祐中 醫師　　　主持人簽章:

學歷：

- 國立台灣大學醫學系醫學士
- 國立台灣大學臨床醫學研究所醫學碩士班肄業

經歷：

- 台大醫院內科住院醫師、感染科總醫師
- 台大愛滋病防治中心專案主治醫師

著作：

1. **Chuang YC**, Wang JL, Chen YC, Chang SC. Characteristics and outcome of community onset septic arthritis in adults. ***J Microbiol Immunol Infect*** (Accepted)
2. Secular Trend of Nosocomial Infection at a Teaching Hospital in Taiwan, 1981-2006 (In Preparation)
