# Supplementary figures and images for: High and Increasing Oxa-51 DNA Load Predict Mortality in Acinetobacter baumannii Bacteremia: Implication for Pathogenesis and Evaluation of Therapy
Source: PLoS One. 2010 Nov 30;5(11):e14133. doi: 10.1371/journal.pone.0014133 (PMC2994729; doi:10.1371/journal.pone.0014133)

# Study design

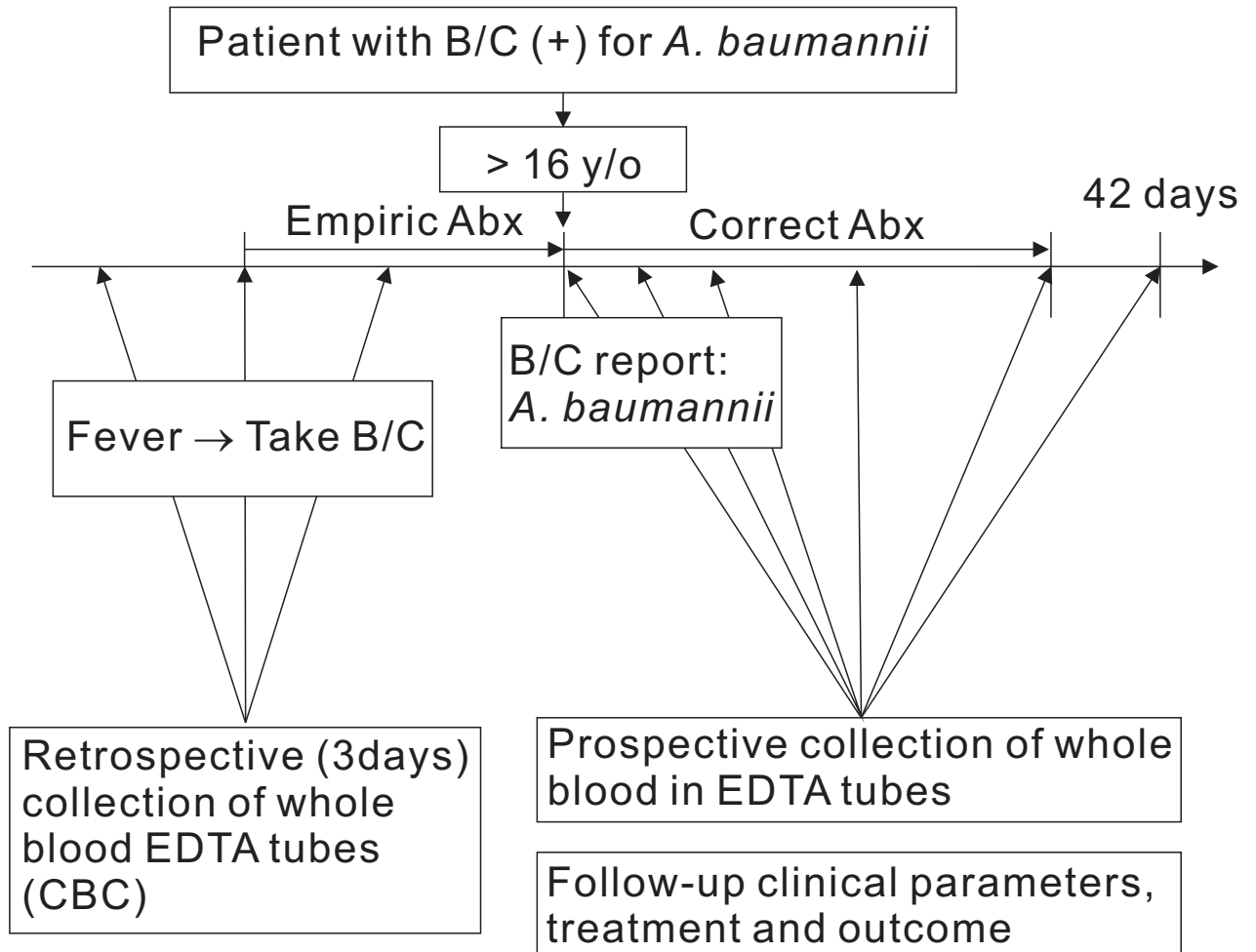

Supplement: Text S2 — Study protocol (0.02 MB PDF) [file pone.0014133.s002.pdf]
