# Supplementary material for: High and Increasing Oxa-51 DNA Load Predict Mortality in Acinetobacter baumannii Bacteremia: Implication for Pathogenesis and Evaluation of Therapy
Source: PLoS One. 2010 Nov 30;5(11):e14133. doi: 10.1371/journal.pone.0014133 (PMC2994729; doi:10.1371/journal.pone.0014133)
Supplement: Text S3 — Quantitative real-time PCR assay for Oxa-51 gene of A. baumannii (0.08 MB DOC) [file pone.0014133.s003.doc]

**Text S3: Quantitative real-time PCR assay for *Oxa-51* gene of *A. baumannii*.**

(A) Schematic diagram of the construct, Oxa-51/pCRII-TOPO, which contains a 431-bp region of the *Oxa-51* gene (position numbers 234 to 664 based on the sequence from GenBank, accession number AJ309734). The relative positions of the primers are shown. The homology of primers and probe with other bacterial sequences was checked by using the gene BLAST™ (<http://blast.ncbi.nlm.nih.gov/Blast.cgi>). Oxa-51/pCRII-TOPO was constructed by PCR amplification of *Oxa-51* gene with primers Oxa-51F234A (5'-TGCTTCGACCTTCAAAATGC-3') and Oxa-51R664B (5'-TAGGGGTTGGTGAAAAACGC-3') and template DNA extracted from a previously reported *A. baumannii* isolate 2003I053 and cloning into pCRII-TOPO [21,32]. (B) Relationship between the input *Oxa-51* DNA copies (5 to 5 x 10^8^ copies) and threshold cycle (CT) number of the assay. (C) Relationship between the CFUs of *A. baumannii* diluted in whole blood and the *Oxa-51* DNA copy number detected by the *Oxa-51* real-time PCR assay. *A. baumannii* isolate (2003I053) [32] was grown from a single colony in Mueller-Hinton broth overnight and was serially diluted 10-fold in normal saline. Fifty microliters of each dilution was plated onto sheep blood agar plates, and the plates were incubated at 37°C overnight to determine the CFU numbers. Increasing amounts of *A. baumannii* (9 x 10^1^ to 9 x 10^6^ CFUs) were spiked with 1 mL whole blood from a healthy donor, and subjected to DNA extraction and real-time PCR assay. (D) Relationship between the *Oxa-51* DNA copy number detected and known amounts of *A. baumannii*, which was spiked with whole blood from a healthy control and stored at 4°C for 0 to 3 days. Briefly, four aliquots of isolate 2003I053 (8 x 10^4^ CFU each) were each spiked with 1 mL whole blood from a healthy donor, stored at 4°C, and subjected to DNA extraction daily for 4 consecutive days and the real-time PCR assay. One representative experiment of more than two was shown. r: correlation coefficient.
