# Supplementary material for: High and Increasing Oxa-51 DNA Load Predict Mortality in Acinetobacter baumannii Bacteremia: Implication for Pathogenesis and Evaluation of Therapy
Source: PLoS One. 2010 Nov 30;5(11):e14133. doi: 10.1371/journal.pone.0014133 (PMC2994729; doi:10.1371/journal.pone.0014133)
Supplement: Text S4 — Multivariate logistic regression analysis (0.04 MB DOC) [file pone.0014133.s004.doc]

**Text S4: Multivariate logistic regression analysis.**

Basic model-fitting techniques for (1) variable selection, (2) assessment of goodness-of-fit (GOF) and (3) regression diagnostics (e.g., residual analysis, detection of influential cases, and check for multicollinearity) were used in the regression analyses to assure the quality of results.

In the stepwise variable selection procedure, all the univariates, of which the *P* < 0.15, and non-significant covariates with biologic meaning were considered and both the significance levels for entry (SLE) and for stay (SLS) were set to 0.15 or larger. Only those variables observed in more than 10 patients were included in the analysis. The GOF measures (such as estimated area under the receiver operating characteristic (ROC) curve, and the adjusted generalized *R*^2^ for logistic regression model) and GOF tests (such as Deviance GOF test, Pearson chi-squared GOF test, and Hosmer-Lemeshow GOF test) were examined. The variance inflation factor (VIF) was used to detect the potential multicollinearity problem (VIF ≥ 10). In other statistical tests, two-sided *P* ≤ 0.05 was considered statistically significant. Since the sample size in some subgroup analyses was not very large, the covariates with a borderline significant *P* value (0.05 < *P* ≤ 0.1) would be retained in the final regression model. Data were analyzed using Stata software, version 10 (StataCorp, College Station, Texas).
