# Supplementary material for: High and Increasing Oxa-51 DNA Load Predict Mortality in Acinetobacter baumannii Bacteremia: Implication for Pathogenesis and Evaluation of Therapy
Source: PLoS One. 2010 Nov 30;5(11):e14133. doi: 10.1371/journal.pone.0014133 (PMC2994729; doi:10.1371/journal.pone.0014133)
Supplement: Table S1 — Antimicrobial susceptibility profile and antibiotic therapy of each study patient with A. baumannii bacteremia. (0.09 MB DOC) [file pone.0014133.s005.doc]

**Table S1: Antimicrobial susceptibility profile and antibiotic therapy of each study patient with *A. baumannii* bacteremia.**

| Patients | Antimicrobial susceptibility of *A. baumannii* ^a^ | Antibiotics therapy ^b^ |
| --- | --- | --- |
| Nonsurvivors |  |  |
| ID2 | S:MER,SUB;R:GM,AM,CIP,LEV,CEF,CTZ,AZR,TIC | LEV on d0 – d2  IMP on d0 – d2 (death) |
| ID3 | R:GM,AM,CIP,LEV,CEF,CTZ,AZR,TIC,MER,SUB | IMP on d0-d4  COL on d3-d13  CEF on d4-d13  SUB on d5-d13 (death) |
| ID4 | I:SUB;R:GM,AM,CIP,LEV,CEF,CTZ,AZR,TIC,MER | Cefpirome on d0-d1  LEV on d1 (death) |
| ID5 | S:MER,SUB;R:GM,AM,CIP,LEV,CEF,CTZ,AZR,TIC | CTZ on d0 (death) |
| ID9 | S:AM,MER;I:SUB;R:GM,CIP,LEV,CEF,CTZ,AZR,TIC | IMP on d0-d9  CIP on d9-d16  IMP on d16-d24 (death) |
| ID10 | I:SUB;R:GM,AM,CIP,LEV,CEF,CTZ,AZR,TIC,MER | IMP on d0-d4  COL on d2-d4 (death) |
| ID11 | R:GM,AM,CIP,LEV,CEF,CTZ,AZR,TIC,MER,SUB | IMP on d0-d2  LEV on d1-d2 (death) |
| ID12 | S:MER,SUB;R:GM,AM,CIP,LEV,CEF,CTZ,AZR,TIC | CEF on d0-d2  SUB on d2-d10  IMP on d2-d10 (death) |
| ID14 | S:MER;I:SUB;R:GM,AM,CIP,LEV,CEF,CTZ,AZR | TIG on d0-d1  CTZ on d1 (death) |
| ID15 | I:SUB;R:GM,AM,CIP,LEV,CEF,CTZ,AZR,TIC,MER | CIP on d0-d2  CTZ on d2 (death) |
| ID16 | I:MER,SUB;R:GM,AM,CIP,LEV,CEF,CTZ,AZR,TIC | TAZ on d1-d2  MER on d1-d6  SUB on d1-d9  COL on d6-d7  TIG on d7-d9 (death) |
| ID17 | S:SUB;R:GM,AM,CIP,LEV,CEF,CTZ,AZR,TIC,MER | IMP on d0-d6  SUB on d3-d6  TIG on d6-d14 (death) |
| ID21 | S:GM,AM,CIP,LEV,CEF,CTZ,TIC,MER,SUB;R:AZR | LEV on d0  IMP on d0 (death) |
| ID23 | S:GM,AM,CIP,LEV,CEF,CTZ,TIC,SUB;I:MER;R:AZR | TAZ on d0-d3  CTZ on d3-d5  SUB on d3-d14  IMP on d5-d14 (death) |
| ID24 | S:GM,AM,CEF,CTZ,TIC,MER,SUB;I:CIP,LEV;R:AZR | CEF on d0-d1 (death) |
| ID25 | R:GM,AM,CIP,LEV,CEF,CTZ,AZR,TIC,MER,SUB | COL on d0  IMP on d0 (death) |
| ID26 | I:CEF;R:GM,AM,CIP,LEV,CTZ,AZR,TIC,MER,SUB | AZR on d1-d4  COL on d4-d11  AZR on d4-d17  d17 (death) |
| ID27 | R:GM,AM,CIP,LEV,CEF,CTZ,AZR,TIC,MER,SUB | IMP d0-d1 (death) |
| ID33 | R:GM,AM,CIP,LEV,CEF,CTZ,AZR,TIC,MER,SUB | CTZ on d0-d1 (death) |
| ID35 | R:GM,AM,CIP,LEV,CEF,CTZ,AZR,TIC,MER,SUB | TIG on d0-d2  CTZ on d0-d2 (death) |
| ID36 | R:GM,AM,CIP,LEV,CEF,CTZ,AZR,TIC,MER,SUB | IMP on d0-d3 (death) |
| ID42 | S:LEV,CTZ,TIC,MER,SUB;R:GM,AM,CIP,CEF,AZR | CEF on d0-d1  IMP on d1 (death) |
| ID43 | R:GM,AM,CIP,LEV,CEF,CTZ,AZR,TIC,MER,SUB | IMP on d0-d2 (death) |
| ID45 | R:GM,AM,CIP,LEV,CEF,CTZ,AZR,TIC,MER,SUB | CIP on d0-d4  TIG on d2-d4 (death) |
| ID47 | R:GM,AM,CIP,LEV,CEF,CTZ,AZR,TIC,MER,SUB | IMP on d0-d2  SUB on d0-d2 (death) |
| ID48 | R:GM,AM,CIP,LEV,CEF,CTZ,AZR,TIC,MER,SUB | CTZ on d0-d1  IMP on d1-d2 (death) |
| ID49 | R:GM,AM,CIP,LEV,CEF,CTZ,AZR,TIC,MER,SUB | IMP on d0-d2 (death) |
| ID52 | R:GM,AM,CIP,LEV,CEF,CTZ,AZR,TIC,MER,SUB | IMP on d0-d2  COL on d2-d4  CTZ on d3-d4 (death) |
| ID53 | R:GM,AM,CIP,LEV,CEF,CTZ,AZR,TIC,MER,SUB | LEV on d0-d1  SUB on d0-d1 (death) |
| ID56 | R:GM,AM,CIP,LEV,CEF,CTZ,AZR,TIC,MER,SUB | TIG on d2-d12 (death) |
| ID57 | S:AM;R:GM,CIP,LEV,CEF,CTZ,AZR,TIC,MER,SUB | COL on d0-d1 (death) |
| ID58 | R:GM,AM,CIP,LEV,CEF,CTZ,AZR,TIC,MER,SUB | IMP on d0-d3  COL on d3-d7 (death) |
| ID59 | I:SUB;R:GM,AM,CIP,LEV,CEF,CTZ,AZR,MER | IMP on d0-d1 (death) |
| ID60 | S:MER,SUB;R:GM,AM,CIP,LEV,CEF,CTZ,AZR,TIC | IMP on d0-d9  SUB on d2-d9 (death) |
| Survivors |  |  |
| ID1 | S:SUB;I:CEF;R:GM,AM,CIP,LEV,CTZ,AZR,TIC,MER | IMP on d0-d6  SUB d4-d11  TIG on d6-d11  COL on d12-d26  CTZ on d6-d55 |
| ID6 | S:MER,SUB;I:TIC;R:GM,AM,CIP,LEV,CEF,CTZ,AZR | IMP on d1-d3  SUB on d5-d18 |
| ID7 | I:TIC;R:GM,AM,CIP,LEV,CEF,CTZ,AZR,MER,SUB | IMP on d1-d16  SUB on d7-d13 |
| ID8 | R:GM,AM,CIP,LEV,CEF,CTZ,AZR,TIC,MER,SUB | TAZ on d0-d3  COL on d3-d15 |
| ID20 | S:GM,AM,CEF,CTZ,MER,SUB;I:LEV;R:CIP,AZR | TAZ on d0-d2  MER on d2-d15 |
| ID28 | I:SUB;R:GM,AM,CIP,LEV,CEF,CTZ,AZR,TIC,MER | CTZ on d0-d23  SUB on d0-d37  d66 (death) |
| ID31 | S:MER,SUB;R:GM,AM,CIP,LEV,CEF,CTZ,AZR,TIC | MER on d3-d14 |
| ID32 | S:AM,LEV,SUB;I:CIP,CTZ,TIC;R:GM,CEF,AZR,MER | CTZ on d0-d2  TAZ on d2-d3  IMP on d3-d16 |
| ID38 | S:SUB;I:TIC;R:GM,AM,CIP,LEV,CEF,CTZ,AZR,MER | CIP on d0-d3  IMP on d3-d9 |
| ID39 | R:GM,AM,CIP,LEV,CEF,CTZ,AZR,TIC,MER,SUB | IMP on d0-d3  COL on d3-d9  TIG on d3-d14  d66 (death) |
| ID40 | S:SUB;I:TIC;R:GM,AM,CIP,LEV,CEF,CTZ,AZR,MER | CIP on d0-d2  AM on d0-d2  COL on d2-d16  d72 (death) |
| ID41 | S:SUB;R:GM,AM,CIP,LEV,CEF,CTZ,AZR,TIC,MER | CTZ on d0-d16  SUB on d2-d16 |
| ID46 | S:AM;R:CIP,LEV,CEF,CTZ,AZR,TIC,MER,SUB | CEF on d0-d4  COL on d4-d17 |
| ID50 | S:CTZ,SUB;I:AM,LEV,CEF;R:GM,CIP,AZR,MER | AM on d0-d12  SUB on d3-d22  CTZ on d14-d22  d121 (death) |
| ID54 | R:GM,AM,CIP,LEV,CEF,CTZ,AZR,TIC,MER,SUB | CEF on d0-d3  TIG on d2-d16  CIP on d3-d15  d45 (death) |
| ID61 | S:GM,AM,CEF,CTZ,TIC,MER,SUB;R:CIP,LEV,AZR | CIP on d0-d1  TAZ on d1-d3  CTZ on d4-8  MER on d8-d22 |
| ID62 | S:GM,AM,CIP,LEV,CEF,CTZ,SUB;R:AZR,TIC,MER | SUB on d0-d2  CTZ on d0-d23  CIP on d2-d23 |

^a^ S, susceptible; R, resistant; I, intermediate. GM, gentamicin; AM, amikacin; CIP, ciprofloxacin; LEV, levofloxacin;

CEF, cefepime; CTZ, ceftazidime; AZR, aztreonam; TIC, ticarcillin/clavulanate; MER, meropenem; SUB, ampicillin/sulbactam; IMP; imipenem; TAZ, piperacillin/tazobactam; TIG, tigecycline; COL, colistin.

^b^ Day 0 (d0) was the day on which the first blood sample positive for *A. baumannii* by culture was drawn.
